# Growing Hyperbranched Polymers Using Natural Sunlight

**DOI:** 10.1038/srep02841

**Published:** 2013-10-08

**Authors:** Jun-Jie Yan, Jiao-Tong Sun, Ye-Zi You, De-Cheng Wu, Chun-Yan Hong

**Affiliations:** 1 CAS Key Laboratory of Soft Matter Chemistry, Department of Polymer Science and Engineering, University of Science and Technology of China, Hefei, 230026, Anhui, P. R. China; 2 Beijing National Laboratory for Molecular Sciences, State Key Laboratory of Polymer Physics & Chemistry, Institute of Chemistry, Chinese Academy of Sciences, Beijing 100190, China

## Abstract

In nature, a sapling can grow into a big tree under irradiation of sunlight. In chemistry, a similar concept that a small molecule only exposing to sunlight grows into a hyperbranched macromolecule has not been realized by now. The achievement of the concept will be fascinating and valuable for polymer synthesis wherein sunlight is inexpensive, abundant, renewable, and nonpolluting. Herein, we report a new strategy in which small monomers can directly grow into big hyperbranched macromolecule under irradiation of sunlight without any catalyst.

Natural photosynthesis of converting solar energy to chemical energy has inspired many chemists to devote their efforts to mimicking its behavior for synthesizing organic molecules or macromolecules^1^,^2^,^3^,^4^,^5^. As early as 1912, Ciamician recognized that sunlight had the potential to serve as an inexpensive, abundant, renewable, and nonpolluting energy for chemical synthesis^5^. However, most conventional organic molecules lack the ability to absorb visible light, greatly restricting the application of sunlight. Currently, most developed efficient organic photo-syntheses and photo-polymerizations typically require high-intensity UV light^6^. Recently, some significant progresses in photosynthesis are to employ photo-catalysts with good abilities of absorbing visible light for sensitizing organic molecules to achieve photochemical reactions or photo-polymerizations^7^,^8^,^9^,^10^,^11^,^12^,^13^,^14^,^15^,^16^,^17^,^18^. Although these as-developed methods are very efficient for photosynthesis of visible light, expensive photosensitive catalysts limit the application on industrial scales. Hyperbranched polymers are one kind of polymers with dendritic structure^19^,^20^,^21^. A sapling can grow into a big tree under irradiation of sunlight. Can small molecules directly grow into hyperbranched polymers when exposed to sunlight without any catalyst?

Recently, it was reported that thiyl radicals can be generated from thiols directly by sunlight shining at low or ambient temperature^22^,^23^. It suggests that thiyl radical based thiol-yne and thiol-ene reactions can be triggered by direct sunlight instead of the usual need of UV in the presence of photo-initiator. Aminolysis of *N*-acetylhomocysteine thiolactone is a facile method for the introduction of sulfhydryl groups in natural proteins^24^,^25^. The method provides a feasible approach to *in situ* generating active thiol groups. Herein, inspired from nature, we report a novel sunlight-activated thiol-yne and thiol-ene strategy to produce hyperbranched polymers from rationally-designed small molecules. The molecules containing both thiols and alkyne or alkene groups are *in situ* generated from ring-opening of alkyne or alkene -containing thiolactone monomers by amino monomers. Then the thiols are activated into thiyl radicals under irradiation of sunlight, and the thiyl radicals react with alkyne or alkene groups to obtain the hyperbranched polymers.

## Results

**Figure 1:**
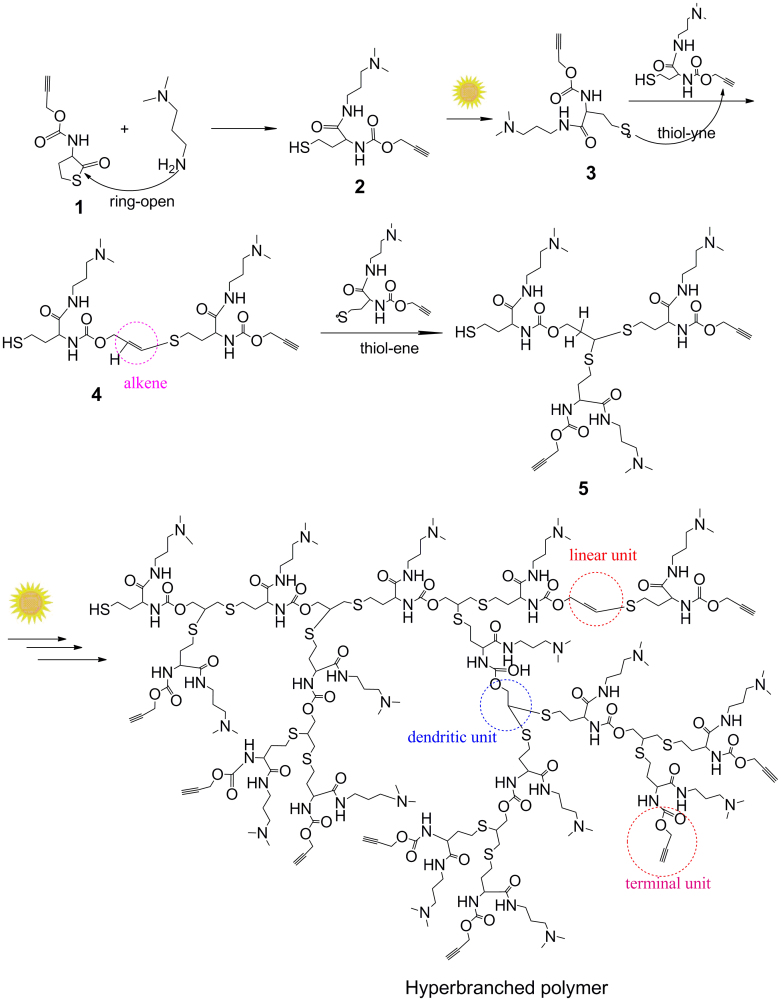
Schematic illustration of growing a hyperbranched polymer via irradiation of the mixture of an alkyne-containing thiolactone and a primary amine by sunlight.

**Figure 2:**
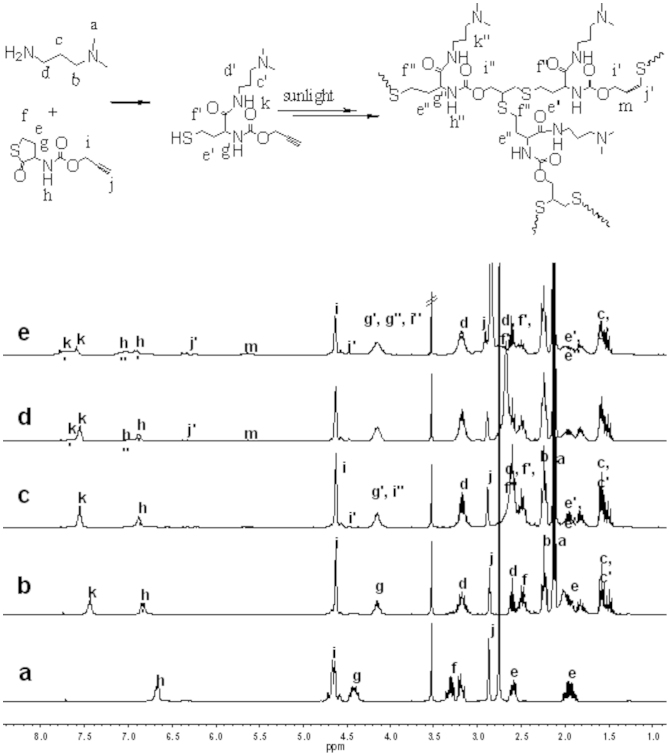
^1^H NMR spectra recorded *in situ* for the reactions of POTC + DMPDA (molar feed ratio is 1:1.25) and thiol-click in 1,4-dioxane-*d_8_* under irradiation of sunlight, the time of sunlight irradiation is 0 min (a), 30 min (b), 110 min (c), 420 min (d) and 900 min (e).

**Figure 3:**
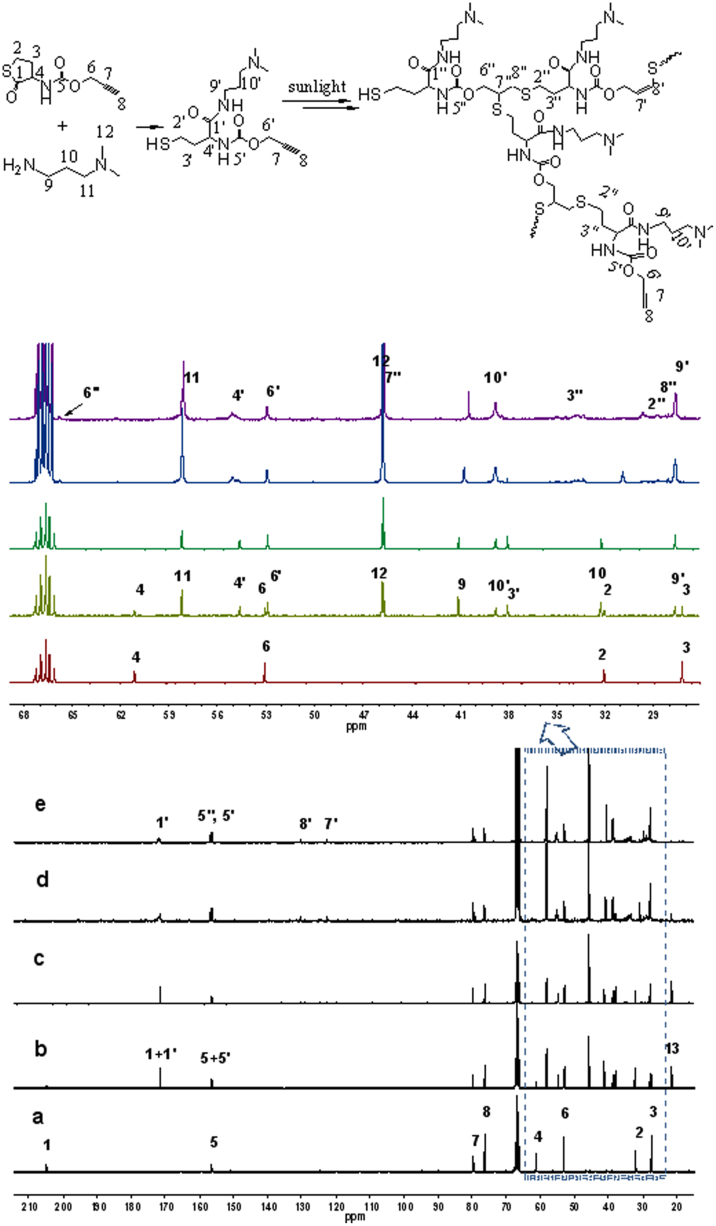
^13^C NMR spectra recorded *in situ* for the reactions of POTC + DMPDA (molar feed ratio is 1:1.25) and thiol-click in 1,4-dioxane-*d_8_* under irradiation of sunlight, the time of sunlight irradiation is 0 min (a), 15 min (b), 110 min (c), 420 min (d) and 900 min (e).

The schematic illustration of growing hyperbranched polymer from small molecules under irradiation of sunlight is proposed in Figure 1. An alkyne-containing thiolactone monomer, prop-2-yn-1-yl(2-oxotetrahydrothiophen-3-yl)carbamate (POTC, **1**), is designed and synthesized as a model molecule. A suitable primary amine, *N,N*-dimethylpropane-1,3-diamine (DMPDA), is selected to ring-open thiolactone group of the POTC monomer to yield a AB_2_-type intermediate(2) containing one *in situ* generated thiol group and one alkyne group. The thiol can be activated into thiyl radical(3) by sunlight^22^,^23^, and the radical attacks an alkyne to form an intermediate(4) with an alkene group, an alkyene group, and a thiol group. Since alkene possesses much higher reactivity than alkyne^26^,^27^,^28^, the thiyl radical is easier to react with the formed alkene to get an intermediate(5). The alternate thiol-yne and thiol-ene click reactions should eventually produce a hyperbranched polymer with a larger number of peripheral alkyne groups and 100% degree of branching if the formed alkene groups always have much higher reactivity than the remained alkyne groups regardless of the factors of concentration and steric hindrance. To verify the proposed mechanism, dynamic ^1^H NMR spectra were *in situ* recorded to monitor polymerization process, and the results were summarized in Figure 2. At an early stage, the formation of the AB_2_ intermediate(2) was detected in Figure 2b and Figure 3. At an initial 2 hours, the nearly alternate thiol-yne and thiol-ene click reactions were proven in Figure 2c by decrease of the signal intensity of alkyne protons (δ = 2.89 ppm) and a negligible signal of the formed alkene protons (δ = 5.6 ppm). With increased reaction time, the detectable signals of the alkene protons indicated the polymerization process did not follow the strict alternated thiol-yne and thiol-ene click reactions (see Figure 2d and Figure 3d). Figure 2e showed that 97% of the *in situ* generated thiols were consumed after irradiation of sunlight for cumulative 15 hours, and the as-produced hyperbranched polymers had an average molecular weight to be 15300 with PDI of 1.62. The degree of branching (DB) of the final product was calculated to be ~ 0.92 according to a formula indicated by the corresponding integral ratios of the proton signals of linear alkene units, terminal alkyne units and dendritic units in Figure 1 and Figure 2e^19b^.

## Discussion

In view of the fact that thiols are easily oxidized when exposed to air, we adopt the aminolysis of thiolactone to *in situ* generate thiols. The nucleophilic ring-opening reaction was monitored via dynamic ^1^H and ^13^C NMR. Figure 2b testified that the aminolysis of thiolactone almost completed within ~ 30 min, due to the corresponding chemical shifts of methine proton of thiolactone (**g**) from 4.41 ppm to 4.13 ppm (**g′**), and amine (**h**) from 6.67 ppm to 6.83 ppm (**h′**). The result can be further verified by the transformation of the lactone unit (chemical shift at 205 ppm) into amide unit (chemical shift at 172 ppm) in ^13^C NMR spectra (see Figure 3) after ring-opening. The aminolysis of thiolactone can be carried out in the presence of primary amines under dark condition, but its rate is a slightly slower than that under irradiation of sunlight. However, secondary and tertiary amines can't ring-open thiolactone even after extended times (e. g., 5 h) (see Supplementary Fig. S10 and S11 online).

**Figure 4:**
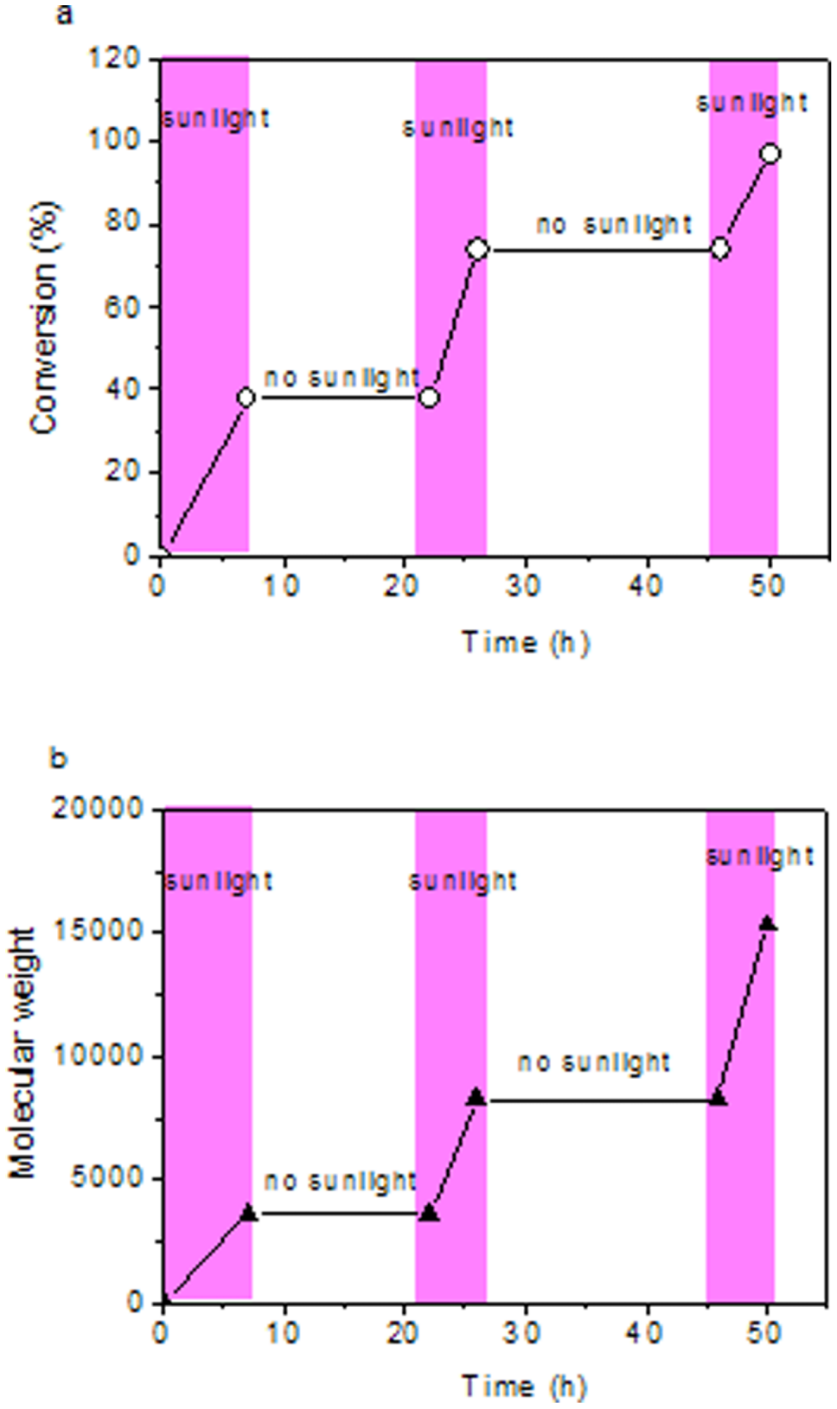
The variations of (A) thiol amount and (B) molecular weight of the formed hyperbranched polymer as a function time with/without irradiation of sunlight.

In this experiment, the generation of thiyl radicals from thiols is of key importance for growing hyperbranched polymers. For identifying whether thiyl radicals are certainly generated from thiols under irradiation of sunlight, some control experiments were carried out. If the radicals can be generated from the employed thiolactone, alkene will form when POTC monomer are directly irradiated by sunlight without adding *N*,*N*-dimethylpropane-1,3-diamine. However, no reaction was detected for POTC monomer exposing to sunlight for 15 h or heating at 70°C for 10 h without the amine (see Supplementary Fig. S12 and S13 online). Therefore, we deduce that the thiyl radical is produced from *in situ* generated thiols when exposed to sunlight, which coincides with the previous findings^22^,^23^. Sunlight is a necessary external stimulus to activate thiols into thiyl radicals, so controlling exposure to sunlight irradiation or not provides a facile and efficient way to manipulate the formation of thiyl radicals, resulting in ‘on/off' function of thiol-yne reaction. Thereby, the consumption of thiols and the growth of hyperbranched polymers can be stopped and recovered. As shown in Figure 3 and Figure 4a, the amounts of the thiols decreased with the increase of irradiation time while it remained unchanged under dark condition after removal from sunlight, and the consumption of thiols continued when sunlight was resumed. About 97% of thiols were consumed after irradiation of sunlight for cumulative 15 h. Similar result was also obtained from the variation of molecular weight of the hyperbranched polymer. Figure 4b show that the weight average molecular weight (M_w_) of the polymer is 3600 with DB of 98% and PDI of 1.32 when the polymerization mixture was irradiated by sunlight for first 7 h, and its M_w_ remained unchanged under dark condition, indicating growth termination of the hyperbranched polymer. The growth of molecular weight recovered after resumption of sunlight, and its M_w_ reached 8200 with DB of 94.8% and PDI of 1.51 under irradiation of next 4 h. The growth stopped in the dark condition for another 20 h, and recovered again after resumption of sunlight and produced a final hyperbranched polymer with a M_w_ to be 15300, DB of 92% and PDI of 1.62 under sunlight irradiation for cumulative 15 h. These results demonstrate that this approach to grow hyperbranched polymers is a controlled process.

In a previous report, the polymers obtained via thiol-yne polymerization under UV irradiation does not have high molecular weight^26^,^27^, which may result from that there are some side reactions in the presence of photo/thermal initiators^25^, but the DB could reach 100% based on thiol-yne polymerization under UV irradiation because thiol-ene reaction is much faster than thiol-yne reaction^27^. In this experiment, there is some residual alkenes in the final hyperbranched polymer (Figure 2e, alkene, δ = 5.0–6.5 ppm), and the DB is determined to be 92 ~ 98% for the obtained polymers. The residual alkenes may result from two factors. The concentration of alkynes is greatly higher than that of alkenes near the end of polymerization as well as higher steric hindrance of the alkenes located inside polymer backbones, reducing differentiation of the reactivity between thiol-yne and thiol-ene reactions.

This method of growing hyperbranched polymers is a versatile approach. General primary amines were suitable for the system, such as 1-(2-aminoethyl)piperazine (AEPZ) and *N*'-(3-aminopropyl)-*N*,*N*-dimethylpropane-1,3-diamine (DMDPTA). The M_w_ of the corresponding hyperbranched polymers are 22000 and 21000 with the DBs of 0.88 and 0.91, PDIs of 2.5 and 2.17 for the polymerization of the mixtures of POTC with AEPZ and DMDPTA, respectively. The as-produced hyperbranched polymers have excellent aqueous solubility, providing prerequisites of functional candidates for bio-applications. Furthermore, this method can be applied in preparing functionalized biocompatible polymers. Amino ended PEG (PEG-NH_2_, M_n_ = 600) was used as a ring-opening agent for thiolactone, and the results verified that PEG-NH_2_ has a similar reactivity as small amino compounds. Viscosity of the reactants obviously increased after irradiation of sunlight for 15 h, and an eventual PEG based hyperbranched polymer had a M_w_ of 12000, DB of 0.95 and PDI of 1.61. Glucosamine was also employed in synthesis of glucose-based hyperbranched polymers using sunlight. The mixture of POTC and glucosamine in DMF was irradiated by sunlight for 20 h. The glucose-based hyperbranched polymer obtained had a M_w_ of 16200, DB of 0.98 and PDI of 3.3.

**Figure 5:**
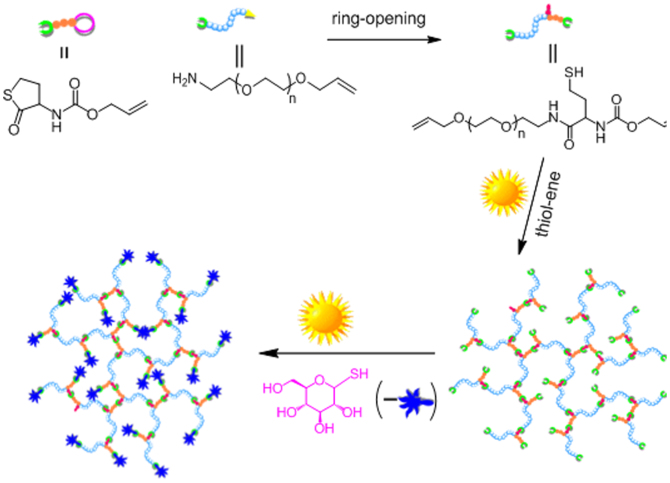
Schematic illustration of growing a hyperbranched polymer from the mixture of *N*-(allyloxy)carbonylhomocysteinethiolactone and amino-PEG under irradiation of sunlight.

**Figure 6:**
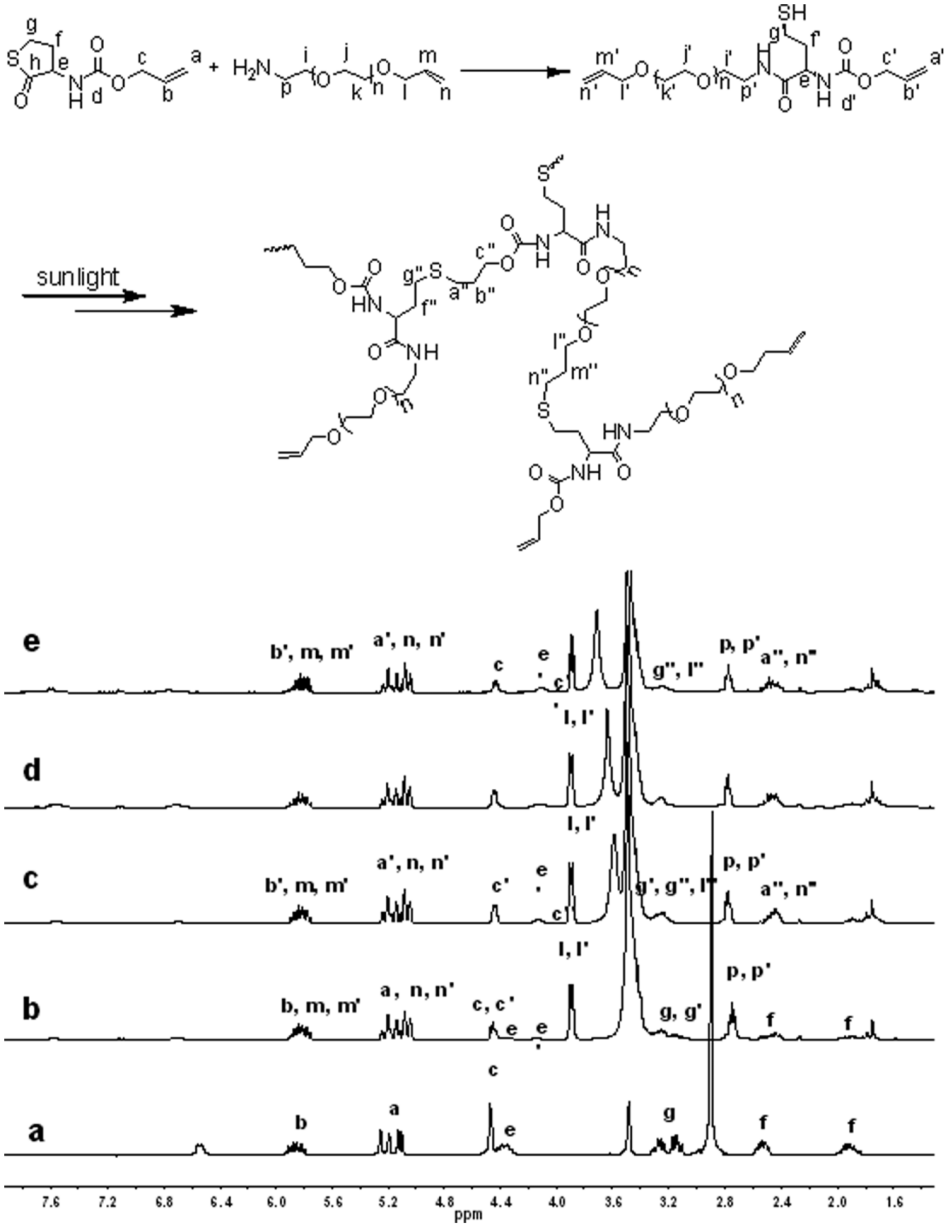
^1^H NMR spectra recorded *in situ* for the reaction of *N*-(allyloxy)carbonylhomocysteine thiolactone with PEG-amine and thiol-ene reaction in 1,4-dioxane-*d_8_* under irradiation of sunlight, the irradiation time is 0 min (a), 30 min (b), 110 min (c), 420 min (d), and 900 min (e).

The method of preparing hyperbranched polymers by sunlight is also applicable for the systems only based on thiol-ene reaction. The mixture of *N*-(allyloxy)carbonylhomocysteine thiolactone (NACHT) and PEG with amine and vinyl groups at two ends exposing to sunlight also produces a hyperbranched polymer, and the approach is clearly illustrated in Figure 5. First, amine of PEG ring-opens *N*-(allyloxy)carbonylhomocysteine thiolactone, yielding an intermediate containing two alkene units and one thiol unit (A_2_B, Figure 5), which can be verified by the NMR results shown in Figure 6. After the ring-opening, the corresponding chemical shifts of methine proton of thiolactone from 4.41 ppm (**e**) to 4.13 ppm (**e′**) (see Figure 6). The result can be further verified by the transformation of the lactone unit (chemical shift at 205 ppm) into amide unit (chemical shift at 172 ppm) in ^13^C NMR spectra (see Supplementary Fig. S14 online), the chemical shift of carbon (**1**) next to sulfur in thiolactone at 32 ppm moving to the carbon (**26**) next to thiol at 20 ppm, the chemical shift of carbon (**2**) in thiolactone at 28 ppm moving to the carbon (**25**) at 38 ppm. All these results indicate the formation of A_2_B intermediate. Subsequently, the thiols transfer into thiyl radicals by sunlight irradiation, triggering thiol-ene reaction to yield hyperbranched polymer with a M_w_ of 24500 and PDI of 5.0. It is clear that the signal for carbon (**25**) next to thiol decreased with the increase of irradiation time (see Supplementary Fig. S14), indicating thiol added onto alkene. On the other hand, the peaks of vinyl unit located at 5.0–6.0 ppm before thiol added onto alkene, some of them moved to the peaks at 1.7–2.0 ppm after thiol added onto alkene (see Figure 6), which further indicated that sunlight triggered thiol-ene reaction. The as-obtained hyperbranched polymer contains many terminal vinyl groups that can be further functionalized via thiol-ene reaction. For example, glucose can be easily grown onto the hyperbranched polymer via sunlight irradiation of the mixture of 1-thio-α-D-glucopyranose (α-GlcSH) and hyperbranched polymer with terminal vinyl groups under argon atmosphere. After irradiation for 12 h, the alkene units are completely consumed. The signals of protons of alkene at 5.9 ppm are completely absent with the appearance of typical proton signals of glucose between 4.1 and 5.6 ppm (see Supplementary Fig. S16 online). After purification via dialysis (MW cut-off: 1000) against water for 2 days, the glucose-functionalized polymer has a molecular weight of 31000.

In summary, we report a novel strategy to grow hyperbranched polymers from small molecules directly under irradiation of sunlight without any photocatalyst. This facile, innovative strategy offers a versatile platform for synthesizing highly functional hyperbranched polymers for bioscience application, and it is green and friendly to environment in a sustainable manner.

## Methods

The syntheses of monomers are shown in Supplementary Materials. All the polymerizations are carried out under irradiation of natural sunlight (weather conditions: sunny; temperature is 25 ~ 36°C; the highest temperature of the reaction mixture is ~ 55°C). A typical polymerization procedure is as following: Prop-2-yn-1-yl (2-oxo tetrahydro thiophen-3-yl)carbamate (50.2 mg, 0.252 mmol) and *N,N*-dimethyl-1,3-propanediamine (32.1 mg, 0.315 mmol) were dissolved in 1,4-dioxane-*d_8_* (550 μL) in a NMR tube filled with argon atmosphere. The tube was sealed and the reaction was then subjected to natural sunlight. The reaction was monitored via ^1^H NMR and ^13^C NMR. The degree of branching (DB) of the as-produced polymer was calculated by the following Formula as originally defined by Fréchet et al.^19^

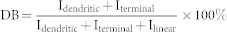


(I denotes the integral value of protons).

## Author Contributions

Y.-Z.Y. and C.-Y.H. conceived and provided the idea of the work together, J.-Y.J. and J.-T.S. performed all the experiments, D.-C.W. analyzed the experiment results, Y.-Z.Y., C.-Y.H. and D.-C.W. wrote the manuscript with assistance from all authors.

## Supplementary Material

Supplementary Information Growing Hyperbranched Polymers Using Natural Sunlight
