# Supplementary material for: Growing Hyperbranched Polymers Using Natural Sunlight
Source: Sci Rep. 2013 Oct 8;3:2841. doi: 10.1038/srep02841 (PMC3792419; doi:10.1038/srep02841)
Supplement: Supplementary Information — Growing Hyperbranched Polymers Using Natural Sunlight [file srep02841-s1.doc]

**Supplementary information**

**Growing Hyperbranched Polymers Using Natural Sunlight**

Jun-Jie Yan^1^, Jiao-Tong Sun^1^, Ye-Zi You^1^*, De-Cheng Wu^2^, Chun-Yan Hong^1^*

^1^CAS Key Laboratory of Soft Matter Chemistry, Department of Polymer Science and Engineering, University of Science and Technology of China, Hefei, 230026, Anhui, P. R. China ^2^Beijing National Laboratory for Molecular Sciences, State Key Laboratory of Polymer Physics & Chemistry, Institute of Chemistry, Chinese Academy of Sciences, Beijing 100190, China

1. **Characterizations**

^1^H NMR, ^13^C-NMR spectra were recorded in 1,4-dioxane-*d_8_* (Alfa-Aesar) or DMSO-*d_6_* (J&K) on a Bruker Avance 400 MHz or Bruker Avance 300 MHz. The NMR spectra were analyzed and processed using MestReNova-6.1.1-6384 software. FT-IR spectra were recorded on a Bruker EQUINOX55 spectrometer using KBr window and performed at room temperature. All sunlight syntheses in this manuscript were carried out under irradiation of natural sunlight (little cloud, ~35 ^o^C). UV source in control experiment was provided by a WFH-204B UV lamp (Shanghai jing secco industrial Co., Ltd). The molecular weights and polydispersities were determined by a gel permeation chromatography (GPC) performed at 35 ^o^C with three linear Styragel columns and a Waters 2414 differential refractive index (RI) detector. DMF was utilized as eluent with flow rate of 1 mL·min^-1^, polystyrenes were used as standard.

1. **Materials**

DL-Homocysteine thiolactone hydrochloride (Aldrich, ≥99%), Propargyl chloroformate (Aldrich, 96%), *N*,*N*-dimethyl-1,3-propanediamine (DMAPA, Alfa Aesar, 99%), *N*'-(3-aminopropyl)-*N*,*N*-dimethylpropane-1,3-diamine (DMDPTA, Aldrich, 99%), 1-(2-Aminoethyl)piperazine (AEPZ, Aldrich, 99%), D–(+)-Glucosamine Hydrochloride (Sigma, ≥99%). *O*-(2-Aminoethyl)polyethylene glycol (PEG-amine, MW ~ 600) was prepared according to the reference with adequate modifications.^1^ Triethylamine was dehydrated over potassium hydroxide overnight, refluxed over paratoluensulfonyl chloride and distilled prior to use. 1,4-dioxane was first refluxed in concentrated hydrochloric acid (10 wt%) for 10 h, then dehydrated in potassium hydroxide overnight, and finally refluxed over sodium and distilled. Other reagents were purchased from Sinopharm Chemical Reagent Co.,Ltd. (SCRC) and used as received.

1. **Synthesis of prop-2-yn-1-yl (2-oxotetrahydrothiophen-3-yl)carbamate (POTC).**

**Scheme S1.** Synthesis of prop-2-yn-1-yl (2-oxo tetrahydro thiophen-3-yl)carbamate.

Prop-2-yn-1-yl (2-oxotetrahydrothiophen-3-yl)carbamate was synthesized according to previous literature.^2^ DL-Homocysteine thiolactone hydrochloride (1.54 g, 10 mmol) was slowly added to a suspension of NaHCO_3_ (4.2 g, 50 mmol) in H_2_O/1,4-dioxane (1/1, 20 mL) and the mixture was stirred for 30 minutes. Propargyl chloroformate (2.37 g, 20 mmol) was added dropwise in 10 min and the mixture was stirred overnight at ambient temperature. The reaction was terminated by diluting with brine (50 mL) and extracted with ethyl acetate (70 mL × 4), the organic phase was collected and dried with anhydrous Na_2_SO_4_, and filtered. The resulting clear solution was concentrated using a rotary evaporator and the residue was purified by a silica column (hexane/ethyl acetate: 1/1) to furnish the product as slight yellow viscous oil, yield is 38%..

**Figure S1**. ^1^H NMR and ^13^C NMR spectrum of prop-2-yn-1-yl (2-oxo tetrahydro thiophen-3-yl)carbamate.

**Figure S2.** FT-IR spectrum of prop-2-yn-1-yl (2-oxo tetrahydro thiophen-3-yl)carbamate.

1. **Synthesis of *N*-(allyloxy)carbonylhomocysteine thiolactone monomer (NACHT).** DL-Homocysteine thiolactone hydrochloride (3.07 g, 0.02 mol) was properly added to a suspension of NaHCO_3_ (8.42 g, 0.1 mol) in H_2_O/1,4-dioxane (1/1, 50 mL) and the mixture was stirred for 30 min. Allyl chloroformate (4.85 g, 0.04 mol) was diluted with 5 mL 1,4-dioxane and added within 20 min, the reaction mixture was stirred for additional 6 h at room temperature. The reaction mixture was terminated with brine (100 mL) and extracted with ethyl acetate (3×150 mL). The organic phase was dried with Na_2_SO_4_, filtered, the filtrate was dried under reduced pressure and purified by column chromatography on silica gel (CH_2_Cl_2_/MeOH: 95/5) to afford colorless viscous oil, yield is 52%.

**Figure S3.** ^1^H NMR and ^13^C NMR spectra of *N*-(allyloxy)carbonylhomocysteine thiolactone in 1,4-dioxane-*d_8_*.

1. **Growing hyperbranched polymer from *N,N*-dimethyl-1,3-propanediamine and** **POTC under irradiation with natural sunlight.**

Prop-2-yn-1-yl (2-oxotetrahydrothiophen-3-yl)carbamate (50.2 mg, 0.252 mmol) and *N,N*-dimethyl-1,3-propanediamine (DMAPA, 32.1 mg, 0.315 mmol) were dissolved in 1,4-dioxane-*d_8_* (550 μL) in a NMR tube filled with argon atmosphere. The tube was sealed and the reaction was then subjected to natural sunlight (weather conditions: sunny, temperature is 25~36 ^o^C; the highest temperature of the reaction mixture is ~55 ^o^C). The reaction was monitored via ^1^H NMR and ^13^C NMR. The degree of branching (DB) of the produced polymer was calculated as originally defined by Fréchet et al.^3^ ($DB=\frac{I(dendritic)+I(ter\min al)}{I(dendritic)+I(ter\min al)+I(linear)}$, *I* denotes the integral values of protons). The DB of the produced polymer was calculated to be about 0.92.^4^ The viscous solution was precipitated in excess of diethyl ether. White viscous solids were obtained by removal of volatile solvent under vacuum (the yield of 69%).

**Figure S4.** ^1^H NMR spectrum of the produced polymer via *N,N*-dimethyl-1,3-propanediamine ring-opening of thiolactone under irradiation with natural sunlight.

$DB=\frac{I_{b}/2+I_{c}/2}{I_{a}+I_{b}/2+I_{c}/2}$, $thiol\text{ }conversion=\frac{I_{a}+I_{b}}{I_{a}+I_{b}/2+I_{c}/2}$.

**Figure S5.** GPC trace of the produced polymer via *N,N*-dimethyl-1,3-propanediamine ring-opening of thiolactone under irradiation with natural sunlight.

**Figure S6.** ^1^H and ^13^C NMR spectra of polymerization before and after being kept under dark for 24 h.

1. **Growing hyperbranched polymer from** [***N*'-(3-aminopropyl)-*N*,*N*-dimethylpropane-1,3-diamine**](http://www.guidechem.com/products/10563-29-8.html) **and** **POTC under irradiation with natural sunlight.** [*N'*-(3-aminopropyl)-*N,N*-dimethylpropane-1,3-diamine](http://www.guidechem.com/products/10563-29-8.html) (DMDPTA, 47.8 mg, 0.30 mmol) and prop-2-yn-1-yl (2-oxotetrahydrothiophen-3-yl)carbamate (44.1 mg, 0.22 mmol) were dissolved in 1,4-dioxane (0.5 mL) in a vial and filled with argon atmosphere. The vial was sealed and exposed to sunlight for 15 h (weather: sunny, temperature is 25~36 ^o^C; the highest temperature of the reaction mixture is ~55 ^o^C). The viscous solution was precipitated in excess of diethyl ether. White viscous solids were obtained via filtration and removal of solvent under vacuum (the yield of 81%). The product was determined to have a molecular weight of Mw = 21000 and a polydispersity of 2.17 via GPC analysis. DB is calculated to be about 0.91 according to ^1^H NMR spectrum.

**A**

**B**

**Figure S7.** (A) ^1^H NMR spectrum of the produced polymer via [*N'*-(3-aminopropyl)-*N,N*-dimethylpropane-1,3-diamine](http://www.guidechem.com/products/10563-29-8.html) ring-opening of thiolactone in DMSO-*d_6_* (* assigns to diethyl ether). (B) ^1^H NMR spectrum of the produced polymer via [1-(2-aminoethyl)](http://www.guidechem.com/products/10563-29-8.html) piperazine ring-opening of thiolactone in DMSO-*d_6_* (*assigns to diethyl ether).

1. **Growing hyperbranched polymer from** [**1-(2-aminoethyl)**](http://www.guidechem.com/products/10563-29-8.html)**piperazine** **and** **POTC under irradiation with natural sunlight.**

[1-(2-Aminoethyl)](http://www.guidechem.com/products/10563-29-8.html)piperazine (AEPZ, 40.2 mg, 0.311 mmol) and prop-2-yn-1-yl (2-oxotetrahydro thiophen-3-yl)carbamate (41.7 mg, 0.210 mmol) were dissolved in 1,4-dioxane (0.5 mL) in a vial filled with argon atmosphere. The tube was sealed with lid and exposed to sunlight (conditions: sunny, temperature is 25~36 ^o^C; the highest temperature of the reaction mixture is ~55 ^o^C) for 15 h. Then the slight yellow mixture was precipitated in excess of diethyl ether, and light yellow viscous solid with a yield of 79% was obtained via filtration and removal of volatile solvent under vacuum. And the product was determined to have a molecular weight of Mw = 22000 and a polydispersity of 2.50 via GPC analysis. DB is calculated to be about 0.88 according to ^1^H NMR spectrum.

1. **Growing hyperbranched polymer from *O*-(2-aminoethyl)polyethylene glycol (PEG-amine)** **and** **POTC under irradiation with natural sunlight.**

*O*-(2-Aminoethyl)polyethylene glycol (PEG-amine, 221.2 mg, 0.369 mmol) and prop-2-yn-1-yl (2-oxotetrahydrothiophen-3-yl)carbamate (43.1 mg, 0.217 mmol) were dissolved in 1,4-dioxane (0.5 mL) in a vial filled with argon atmosphere. The tube was sealed with lid and exposed to sunlight for 15 h (weather: sunny, temperature is 25~36 ^o^C; the highest temperature of the reaction mixture is ~55 ^o^C). Then the slight yellow mixture was precipitated in excess of diethyl ether， and light yellow viscous liquids with yield of 73% was obtained via filtration and removal of solvent under vacuum. And the product was determined to have a molecular weight of 19000 and a polydispersity of 1.61 via GPC analysis. DB is calculated to be about 0.95 according to ^1^H NMR spectrum.

**Figure S8.** ^1^H NMR spectrum of the produced polymer via *O*-(2-aminoethyl)polyethylene glycol ring-opening of thiolactone in DMSO-*d_6_* (*assigns to diethyl ether).

1. **Growing hyperbranched polymer from glucosamine and** **POTC under irradiation with natural sunlight.**

**Figure S9.** ^1^H NMR spectrum of the produced polymer via glucosamine ring-opening of thiolactone in DMSO-*d_6_* (*assigns to diethyl ether).

D–(+)-Glucosamine hydrochloride (135.6 mg, 0.628 mmol), prop-2-yn-1-yl (2-oxotetrahydrothiophen-3-yl)carbamate (57.6 mg, 0.289 mmol) and triethylamine (123.1 mg, 0.82 mmol) were dissolved in DMF (1.0 mL) in 3.5 mL a quartz cuvette (Q-14, 45 mm ×12.5 mm ×12.5 mm, Yixing JingKe Optical Instrument Co., Ltd.) and filled with argon atmosphere. The cuvette was sealed with lid and exposed to sunlight for 20 h (weather: sunny, temperature is 25~36 ^o^C; the highest temperature of the reaction mixture is ~55 ^o^C). Then the yellow mixture was precipitated in excess of diethyl ether, and orange viscous oil with a yield of 83% was obtained via filtration and removal of solvent under vacuum. And the product was determined to have a molecular weight of 16200 and a polydispersity of 3.30 via GPC analysis. DB is calculated to be about 0.98 according to ^1^H NMR spectrum.

1. **Ring-opening with secondary.**

Prop-2-yn-1-yl (2-oxo tetrahydro thiophen-3-yl)carbamate (49.6 mg, 0.249 mmol) and diethylamine (26 mg, 0.35 mmol) were dissolved in 1,4-dioxane (550 μL) in a NMR tube filled with argon atmosphere. The tube was sealed and the reaction was then subjected to irradiation of natural sunlight for accumulative 5 h, subsequently, ^1^H NMR spectrum was recorded.

**Figure S10.** ^1^H NMR spectrum of reaction of prop-2-yn-1-yl (2-oxotetrahydro thiophen-3-yl)carbamate and diethylamine.

1. **Ring-opening with secondary and tertiary amine.**

prop-2-yn-1-yl (2-oxo tetrahydro thiophen-3-yl)carbamate (49.6 mg, 0.249 mmol) and triethylamine (35 mg, 0.35 mmol) were dissolved in 1,4-dioxane (550 μL) in a NMR tube filled with argon atmosphere. The tube was sealed and the reaction was then subjected to irradiation of natural sunlight for accumulative 5 h, subsequently, ^1^H NMR spectrum was recorded.

**Figure S11.** ^1^H NMR spectrum of reaction of prop-2-yn-1-yl (2-oxotetrahydro thiophen-3-yl)carbamate and triethylamine.

**Figure S12.** ^1^H NMR spectrum of prop-2-yn-1-yl (2-oxotetrahydro thiophen-3-yl)carbamate in dioxane after irradiation with nature sunlight for accumulative 15 h.

**Figure S13.** ^1^H NMR spectrum of prop-2-yn-1-yl (2-oxo tetrahydro thiophen-3-yl)carbamate in dioxane after keeping at 70 ^o^C for 10 h.

1. **Growing hyperbranched polymer from NACHT and PEG with amine and alkene ends.** NACHT (39.8 mg, 0.198 mmol) and amine-PEG-vinyl (60.2 mg, ~ 0.20 mmol) were dissolved in 1,4-dioxane-*d_8_* (550 μL) in a NMR tube filled with argon atmosphere. The tube was sealed and every interval vortexing was implied to ensure the homogeneity of the mixture, the reaction was subjected to sunlight for accumulative 15 h (weather: sunny, temperature is 25~36 ^o^C; the highest temperature of the reaction mixture is ~55 ^o^C). Then the mixture was precipitated in excess of diethyl ether, and viscous solid was boatined with a yield of 81% via filtration and removal of the volatiles under vacuum. And the product was determined to have a molecular weight of 24500 and a polydispersity of 5.0 via GPC analysis

**Figure S14**. Dynamic ^13^C NMR spectrum of aminolysis of compound NACHT with PEG-amine and consecutive thiol-ene reaction. (The beginning of the reaction was set at the time when PEG-amine was added, t = 0 min.)

**Functionalizing hyperbranched polymer with glucose.** PEG based hyperbranched polymer (25 mg, 5.1× 10^-3^ mmol, [alkene] ~ 0.05 mmol), α-GlcSH (30 mg, 0.15 mmol) were dissolved in the mixed solvents of 1,4-dioxane and DMF (0.4 mL, 1/1, v/v), and added into NMR tube under argon atmosphere, after vortexing for 1 min, it was subjected to sunlight radiation for accumulative 12 h (weather: sunny, temperature is 25~36 ^o^C; the highest temperature of the reaction mixture is ~55 ^o^C). The mixture was purified by precipitating into excess of diethyl and dialysis (MW cut-off: 1000) against water for 2 days, vicious solids were obtained after removal of water, the yield is 46%.

**Figure S15**. FT-IR spectra of (a) *N*-(allyloxy)carbonylhomocysteine thiolactone (b) PEG based hyperbranched polymer and (c) glucose functionalized hyperbranched polymer.

**Figure S16**. ^1^H NMR spectra of PEG based hyperbranched polymer before and after decorated with glucose.

**References**

1. Bawendi, M. G.; Liu, W. H.; Greytak, A. B.; Lee, J.; Wong, C. R.; Park, J.; Marshall, L. F.; Jiang, W.; Curtin, P. N.; Ting, A. Y.; Nocera, D. G.; Fukumura, D.; Jain, R. K. *J. Am. Chem. Soc.***132,** 472-483 (2010).
2. Espeel, P.; Goethals, F.; Du Prez, F. E. *Journal of the American Chemical Society* **133**, 1678-1681 (2011).
3. Hawker, C. J.; Lee, R.; Frechet, J. M. J. *J. Am. Chem. Soc* **113,** 4583-4588 (1991).
4. Han, J.; Zhao, B.; Gao, Y.; Tang, A.; Gao, C. *Polymer Chemistry* **2**, 2175-2178 (2011).
